# RBM4 regulates cellular senescence via miR1244/SERPINE1 axis

**DOI:** 10.1038/s41419-023-05563-z

**Published:** 2023-01-13

**Authors:** Luning Wang, Xiaohong Zhang, Junxiu Sheng, Lei Chen, Lili Zhi, Qianqian Zheng, Yangfan Qi, Linlin Wang, Jinrui Zhang, Jinyao Zhao, Yang Wang, Shu-Xin Liu, Ming-Zhong Sun, Wenjing Zhang

**Affiliations:** 1 Institute of Cancer Stem Cell, Dalian Medical University, Dalian, 116044 China; 2 Department of Biotechnology, College of Basic Medical Sciences, Dalian Medical University, Dalian, 116044 China; 3 Department of Radiation Oncology, First Affiliated Hospital of Dalian Medical University, Dalian, 116044 China; 4 Department of Nephrology, Dalian Municipal Central Hospital, Dalian Medical University, Dalian, 116033 China

**Keywords:** Non-small-cell lung cancer, Senescence

## Abstract

Cellular senescence serves as a powerful tumor suppressing mechanism that inhibits the proliferation of cancer cells bearing oncogenic mutations at the initial stage of cancer development. RNA-binding proteins (RBPs) play important roles in cancer progression and treatment through distinct functions. However, functions and mechanisms of RNA binding proteins in regulating senescence remain elusive. Here we reported that the RNA binding protein RBM4 contributed to cellular senescence. Depletion of RBM4 induced senescence in different types of cells, including multiple cancer cells. Meanwhile, RBM4 ablation inhibited cancer cell progression both in vitro and in vivo. Specifically, knockdown of RBM4 significantly increased the level of SERPINE1, a known promoter of senescence, thereby inducing the senescence of lung cancer cells. Mechanistically, miR-1244 bound to the 3ʹ-UTR of SERPINE1 to suppress its expression, whereas depletion of RBM4 reduced the level of miR-1244 by promoting the degradation of primary miR-1244 transcripts (pri-miR1244), thus increasing the expression of SERPINE1 and inducing subsequent senescence. Moreover, either SERPINE1 inhibitor or miR-1244 mimics attenuated the RBM4 depletion-induced senescence. Altogether, our study revealed a novel mechanism of RBM4 in the regulation of cancer progression through controlling senescence, providing a new avenue for targeting RBM4 in cancer therapeutics.

## Introduction

Lung cancer is one of the most commonly diagnosed cancer and the leading cause of cancer death, with an estimated 2.2 million new cancer cases and 1.8 million deaths in 2020 [1]. Treatment options for lung cancer include surgery, chemotherapy, radiation, immunotherapy and targeted therapies. Despite there has been significant progress in the treatment of lung cancer in the past 10 decades, the incidence and mortality for lung cancer is still high. Thus, there is an urgent need to understand the molecular mechanisms of occurrence and development of lung cancer and to explore novel strategies for lung cancer.

Cellular senescence is a state of irreversible cell cycle arrest triggered by diverse forms of cellular stress inducers such as telomere shortening caused by extensive passaging, activation of oncogenes and chemotherapeutics. Senescent cells usually exhibit distinct phenotypes, including an enlarged and flattened morphology, accumulation of senescence-associated heterochromatic foci and elevated senescence-associated β-galactosidase (SA-β-gal) activity [2–6]. Cellular senescence is often considered as an intrinsic tumor suppressor mechanism in the early stage of tumorigenesis [7, 8]. Cancer cells can mount a senescence response as well. Most anticancer treatment, such as chemotherapy and radiation, has been shown to induce senescence in patients with neoplasia [8]. Pro-senescence therapy has been proposed as a promising strategy for cancer treatment [9].

RNA binding proteins (RBPs) are a class of highly conserved proteins that involved in various aspects of the metabolism of RNA, including alternative splicing, polyadenylation, modification, stability, and translation of mRNA, as well as miRNAs and circular RNA processing [10, 11]. There is growing evidence that dysregulation or dysfunction of RBPs can lead to human diseases, including cancers [11]. RBPs are involved in multiple cancer-associated phenotypes including proliferation, apoptosis, senescence, migration, invasion, and angiogenesis [12, 13]. As a member of RBPs, RBM4 is a multifunctional RNA-binding protein mainly involving in regulating alternative splicing and mRNA translation [14–17]. RBM4 was generally considered as a potential tumor suppressor to inhibit cancer progression through regulating alternative splicing of cancer-related genes [16, 17]. In addition to splicing regulation, RBM4 can also translocate to the cytoplasm [18], where it participates in the control of cap-dependent translation and mRNA turnover [14, 19, 20]. Here we describe the role of RBM4 in cellular senescence. Mechanistically, downregulation of RBM4 post-transcriptionally activated SERPINE1 through reducing the level of miR-1244, thereby inducing the senescence of cancer cells. Overall, our study revealed a novel mechanism of RBM4 in the regulation of tumorigenesis through controlling senescence, providing a new avenue for targeting RBM4 in cancer therapy.

## Materials and methods

### Cell culture

Cell lines used in this study were obtained from the American Type Culture Collection (ATCC) and cultured under standard culture conditions in culture medium recommended by the ATCC. HEK-293T and Hela cells were maintained in DMEM with 10% FBS. NCI-H1299 and 786O cells were cultured with RPMI-1640 media supplemented with 10% FBS, while A549 cells were maintained using F12-K media supplemented with 10% FBS. MRC5 cells were maintained in MEM with 10% FBS. HFL1 were maintained using F12-K media supplemented with 10% FBS. HCT116 cells were cultured with McCoy’s 5a supplemented with 10% FBS. HeyA8 cells were cultured in RPMI-1640 medium supplemented with 15% FBS.

### Western blot

Cells were washed with cold PBS and then resuspended in RIPA lysis buffer containing 1 mM Na_3_VO_4_, 1 mM Cocktail and 1 mM PMSF. The cell lysates were centrifuged at 12000 rpm for 15 min and the protein concentration was measured using BCA protein assay kit. Equal amounts of total protein were mixed with loading buffer and denatured for 5 min at 95 °C. Proteins were loaded onto SDS-polyacrylamide gels and transferred to nitrocellulose membrane. The membranes were blocked with 4% fat-free milk and incubated with primary antibodies at 4 °C overnight. After PBST washes for 3 times, The membranes were incubated with secondary antibody for 1 h at room temperature. Protein bands were visualized with the ECL kit (GE Healthcare). The following antibodies were used: RBM4 (Proteintech, 11614-1-AP), Phospho-Rb (Ser795, Cell signaling, #9301), FLAG (Sigma 1804), VINCULIN (Proteintech 66305-1-Ig), GAPDH (Proteintech 60004-1-Ig), CDK2 (Cell signaling, #78B2), CDK4 (Cell signaling, #D9G3E), CDK6 (Cell signaling, #DCS83), Cyclin D3 (Cell signaling, #DCS22), SERPINE1 (Proteintech, 13801-1-AP) and tubulin (Abcam, ab11316).

### RNA isolation and RT-qPCR

RNA was extracted from cells using Trizol (Invitrogen) according to the manufacturer’s instructions. Briefly, A549 or H1299 cells were lysed with 1 ml Trizol reagent. Transfer the cell lysate to a 1.5 mL centrifuge tube and add 0.2 mL of chloroform. Mix it thoroughly and incubate at room temperature for 5 min. Centrifuge the mixture at 12,000 × *g* for 15 min at 4 °C and transfer the aqueous phase to a fresh tube. RNA were precipitated by adding 0.5 mL of isopropanol followed by incubation for 5 min at room temperature. Centrifuge the RNA precipitate at 12,000 × *g* for 10 min at 4 °C. Remove the supernatant and wash the RNA pellet once with 1 mL of 75% ethanol. Air-dry the RNA pellet for 10 min and resuspend in RNAse free water. Then, RNA samples were treated with DNase I (Promega M6101) at 37 °C for 30 min to remove genomic DNA contaminants. Total RNA (2 µg) was then reverse-transcribed with PrimeScript RT reagent kit (Takara) with random primer. The real-time PCR were performed using the Maxima SYBR Green qPCR Master Mix (Thermo Scientific) and a 7500 real-time PCR system (Life Technologies) according to manufacturer’s instructions. Gene expression was determined by using the 2^−ΔΔCt^ method. The primers listed in Supplementary Table.

### β-gal staining assay

For senescence-associated β-gal staining assay, H1299, A549, HFL1, MRC5, HeyA8, HCT116, HeLa or 786 O cells were infected with lentiviral-based control sh, RBM4 sh1 or RBM4 sh2 for 24 h. Subsequently, 48 h post infection, cells were plated in 6-well plates. The next day, medium was removed and cells were washed with PBS for 3 times. Then cells were fixed and stained using cell senescence β-Galactosidase staining Kit (Beyotime, C0602).

### EdU assays

The proliferation of H1299 and A549 cells were detected by using Cell-Light EdU Apollo488 In Vitro Kit (RiboBio) according to the manufacturer’s protocol. Briefly, cells were incubated with 50 μM EdU for 2 h before fixation with 4% paraformaldehyde. Cells were treated with 2 mg/mL glycine for 5 min followed by PBS wash. Cells were permeabilized by 0.5% Triton X-100 for 10 min. After extensive washing with PBS, cells were stained with 1× Apollo reaction solution for 30 min. Then, Cell nuclei were stained with 5 μg/mL Hoechst 33342 for 30 min. Cells were photographed using a fluorescence microscope (Leica Mi8) and the number of EdU-positive cells were counted.

### Colony formation assays

For the colony formation assay, cells (1 × 10^3^ cells/well) were seeded in the 10-cm dishes and incubated at 37 °C in humidified incubator. When cell colonies were clearly identifiable, the colonies were fixed with methanol and stained with crystal violet. After washing with water, cell colonies were photographed for statistics.

### Luciferase assay

H1299 cells were transfected with pmirGLO plasmid containing SERPINE1 3ʹUTR and miR-1244 mimic or miR NC for 24 h. The luciferase activities were measured following dual luciferase reporter assay detection kit (Promega Corporation, USA).

### Xenograft assays

The Institutional Animal Care and Use Committee of Dalian Medical University approved the experimental protocols performed on the animals. H1299 cells with doxycycline-inducible depletion of RBM4 (5 × 10^6^) were injected subcutaneously into the abdomen side of 6-week-old BALB/c nude mice. Macroscopic observation and tumor volume measurements were performed every three days. When tumors reached the volume of approximately 50 mm^3^, the mice were randomly and blindly divided into two groups (eight per treatment group) and were fed with water in the presence or absence of doxycycline. The tumor growth was monitored for thirty days. The xenograft tumor was calculated according to the formula: V = length × width^2^/2. Mice were euthanized and excised tumors were weighed.

### Statistical analysis

All data are presented as the means ± SD from at least three independent experiments. Statistical significance for each experiment was established by two-tailed unpaired or paired *t* test, and one-way or two-way ANOVA, as appropriate. Statistical analyses were performed using Prism 9 (Graph Pad).

## Results

### Depletion of RBM4 induces cellular senescence

**Fig. 1:**
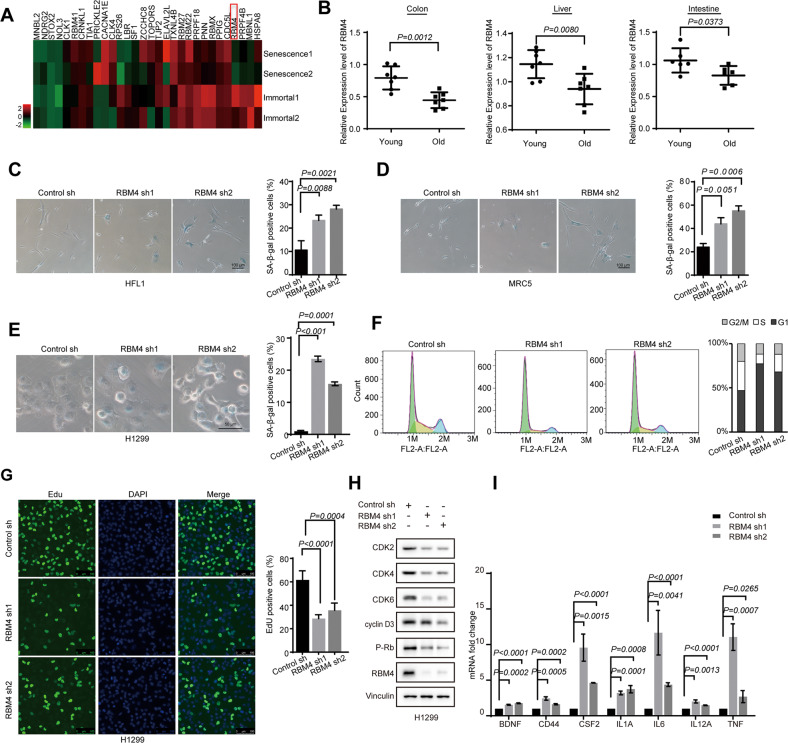
Depletion of RBM4 induces cellular senescence. **A** The levels of selected RNA binding proteins in senescent and immortal cells were analyzed using GSE60340 datasets, and presented by heatmap. **B** Colons, livers, and intestines from young (2 months) and old (24 months) C57BL/6 female mice were applied to examine the mRNA levels of RBM4 using RT-qPCR. **C** Changes of SA-β-gal activity in RBM4 knocking down or control human fibroblasts HFL1 cells. Data represent the percentage of cells staining positive for SA-β-gal ± SD (*P* values were determined by One-way ANOVA with Dunnett multiple comparisons). **D** Changes of SA-β-gal activity in RBM4 knocking down or control human fibroblasts MRC5 cells. Data represent the percentage of cells staining positive for SA-β-gal ± SD (*P* values were determined by One-way ANOVA with Dunnett multiple comparisons). **E** β-gal staining of H1299 cells with stable knockdown of RBM4. Three experiments were carried out with mean ± SD of β-gal positive cells plotted (*P* values were determined by One-way ANOVA with Dunnett multiple comparisons). **F** Cell cycle analysis calculated the distribution of the cells in G1, S and G2/M phases. **G** The proliferative abilities of stably RBM4-depleted H1299 cells were measured with an EdU staining assay. Three experiments were conducted, with mean ± SD of percentage of EdU-positive cells plotted (*P* values were determined by One-way ANOVA with Dunnett multiple comparisons). **H** The protein levels of RBM4, CDK2, CDK4, CDK6, cyclin D3 and p-Rb in H1299 cells with RBM4 depletion were examined using a western blot assay. **I** The mRNA expression levels of senescence associated pro-inflammatory genes including BDNF, CD44, interleukin 1 A, 6, 12 A, TNF in RBM4-depleted H1299 cells were examined by RT-qPCR. *P* values were determined by One-way ANOVA with Dunnett multiple comparisons.

Since cellular senescence can be triggered by distinct factors, alterations of RBPs might be also involved in this process. However, the role and mechanisms of how RNA binding proteins participate in senescence are still elusive. To conduct an unbiased identification of RBPs in senescence, we analyzed previously published senescence related datasets (GSE60340) [21], and found that the levels of a series of RBPs were changed in H_2_O_2_-induced senescent cells, including MBNL1, CLK4, RBMX, RBM4 and so on (Fig. 1A). Among these potential senescence-related RBPs, we focused on RBM4 because: (1) it is a critical RNA binding protein in cancer progression; (2) it is one of the most downregulated genes in senescent cells. We further investigated whether RBM4 expression is different during aging in vivo. To this end, we extracted RNAs from colon, liver, and intestine tissues of C57BL/6 female mice aged 2 and 24 months. Consistently, all the three kinds of tissues from aged 24 months demonstrated a significant decrease of RBM4 level as compared to that from aged 2 months mice (Fig. 1B).

The aforementioned data suggest that RBM4 might be involved in cellular senescence regulation. We subsequently knocked down RBM4 in HFL1 cells, which are normal human lung fibroblasts. As expected, downregulation of RBM4 indeed induced senescence in HFL1 cells, as judged by the elevation of the number of cells staining positive for SA-β-gal activity (Fig. 1C and Fig. S1A). Moreover, such senescence phenotype was also induced by depletion of RBM4 in MRC5 cells, another human normal lung fibroblast cell line (Fig. 1D and Fig. S1B).

Previously we have shown RBM4 inhibits lung cancer progression, thus we next want to determine whether RBM4 could contribute to senescence in lung cancer cells. Interestingly, we found that knockdown of RBM4 induced the enlarged and flattened alteration of cell morphology, as well as significantly increased SA-β-gal activity (Fig. 1E). In addition, we further evaluated the cell cycle change, and found a G1/S phase arrest, indicating that a cell proliferation arrest was induced by decreased level of RBM4 (Fig. 1F). Consistently, such proliferation inhibition was also validated by measuring the percentage of cells incorporating ethynyldeoxyuridine (EdU) (Fig. 1G). Meanwhile, protein levels of a panel of senescence-related markers were evidently changed accordingly (Fig. 1H). Since senescent cells secret a wide range of chemokines, cytokines, and other proteins, which are termed as SASP. We next determined whether depletion of RBM4 could promote SASP. Importantly, we found that knockdown of RBM4 led to more than two-fold upregulation of the mRNA levels of many senescence associated pro-inflammatory genes, including BDNF, CD44, interleukin 6, TNF and so on (Fig. 1I). Collectively, such RBM4-depletion induced senescence is cell context independent, as knockdown of RBM4 also markedly stimulated senescence in A549 lung cancer cells (Fig. S1C–1G).

### Loss of RBM4 induces senescence and inhibits tumorigenesis in multiple cancer cells

**Fig. 2:**
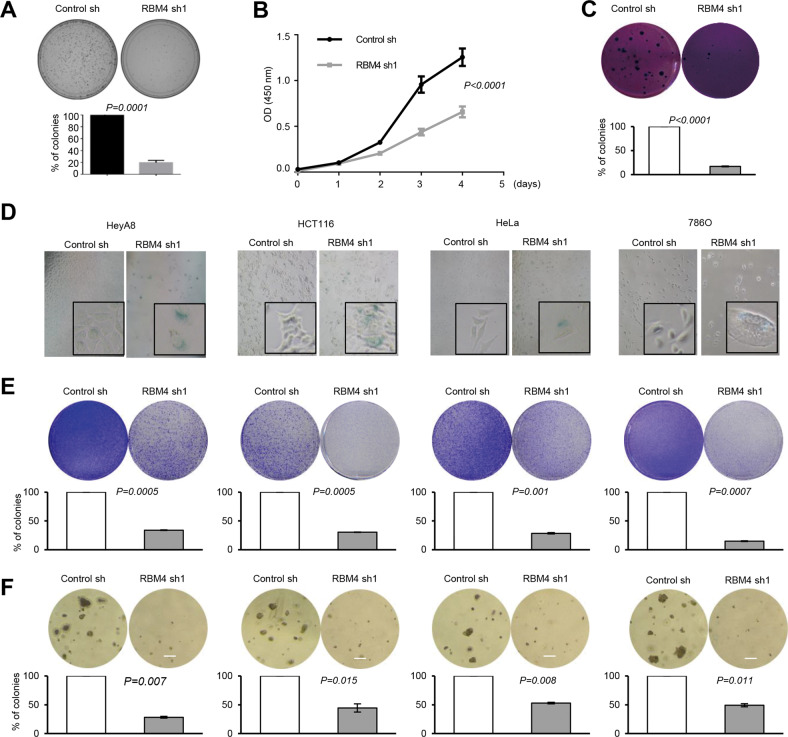
Loss of RBM4 induces senescence and inhibits tumorigenesis in multiple cancer cells. **A** Colony formation assays of RBM4-depleted H1299 cells were performed. **B** Cell viability of RBM4-depleted H1299 cells were examined using CCK8 assay. **C** Soft agar assays of RBM4-depleted H1299 cells were performed. **D** β-gal staining of HeyA8, HCT116, Hela and 786 O cells with stable knockdown of RBM4. **E** Colony formation assays of HeyA8, HCT116, Hela and 786 O cells with stable knockdown of RBM4. **F** Soft agar assays of HeyA8, HCT116, Hela and 786 O cells with stable knockdown of RBM4 were performed.

Senescence has been demonstrated to suppress cancer cell progression, thereby providing an early barrier for tumorigenesis [22, 23]. We next determined whether the senescence induced by RBM4-depletion played crucial roles in tumor suppression. We found that in lung cancer cells, knockdown of RBM4 could not only induce senescence, but also inhibit anchorage dependent or independent growth as judged by colony formation, cell viability, or soft agar assays (Fig. 2A–C and Fig. S2A).

To examine whether the senescence induction is a common role of RBM4, we stably knocked down RBM4 in a panel of human cancer cells, including HeyA8 (ovarian cancer), HCT116 (colon cancer), HeLa (cervical cancer), and 786 O (renal cancer) (Fig. S2B). Importantly, in all cancer cells examined, depletion of RBM4 induced senescence as judged by increased SA-β-gal activity (Fig. 2D). Similarly, knockdown of RBM4 inhibited cancer cell growth and proliferation in all tested cancer cells (Fig. 2E, F). Taken together, depletion of RBM4-induced senescence and tumor suppression is a common role of RBM4 in distinct cancer cells.

### RBM4 ablation promotes senescence to suppress cancer progression in vivo

**Fig. 3:**
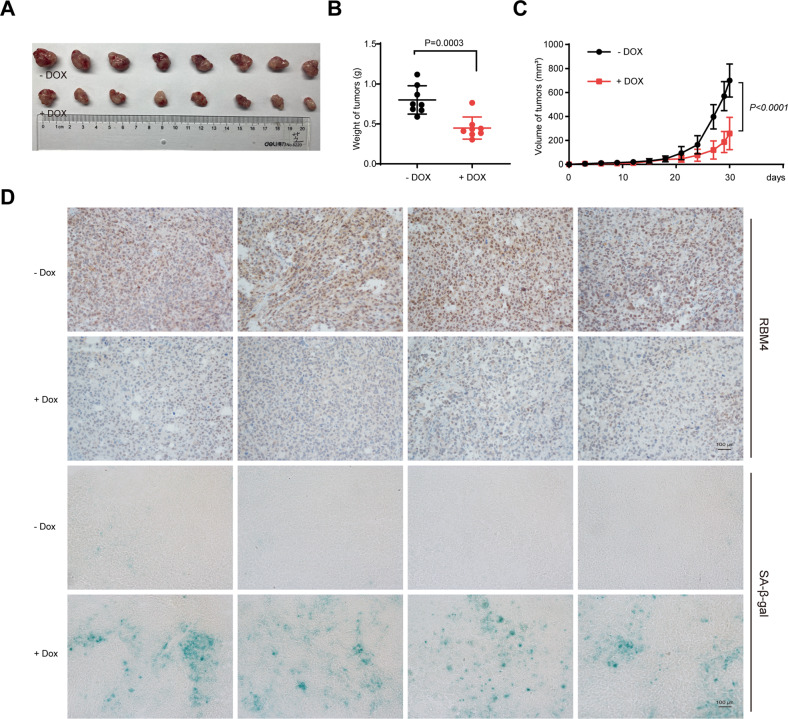
RBM4 depletion promotes senescence to suppress cancer progression in vivo. **A** Xenograft tumors were generated using nude mice subcutaneously injected with H1299 cells with doxycycline-inducible depletion of RBM4. **B** Tumors were weighed and plotted. **C** The average sizes of xenograft tumors were measured every 3 days and plotted (*n* = 8, error bars indicate mean ± SD). **D** Immunohistochemical staining of SA-β-gal, and RBM4 in tumor from RBM4-depleted H1299 cells.

To further assess the role of RBM4 ablation in senescence regulation and cancer suppression in vivo, we subcutaneously inoculated H1299 cells with doxycycline-inducible depletion of RBM4, into the flanks of nude mice. When tumors reached the volume of approximately 50 mm^3^, the mice (eight per treatment group) were fed with water in the presence or absence of doxycycline. The tumor growth was monitored every three days for thirty days, and xenograft tumors were removed for further investigation. Consistent with the in vitro data, cells with doxycycline-inducible knockdown of RBM4 displayed significantly smaller tumors as compared to control cells (Fig. 3A, B). Additionally, the xenograft tumors with inducible-depletion of RBM4 demonstrated a much slower growth rate than controls (Fig. 3C), indicating that depleted RBM4 significantly suppresses cancer progression in vivo.

To validate the role of RBM4 in regulating senescence in vivo, we carried out an immunohistochemistry (IHC) analysis using removed mice tumor samples. Importantly, downregulation of RBM4 dramatically elevated SA-β-gal activity in tumors (Fig. 3D). Altogether, our findings suggest that RBM4 ablation plays a critical role in inducing senescence and suppressing tumor growth both in cultured cancer cells and in a tumor xenograft models.

### Global identification of genes that are regulated by depletion of RBM4 in senescence

**Fig. 4:**
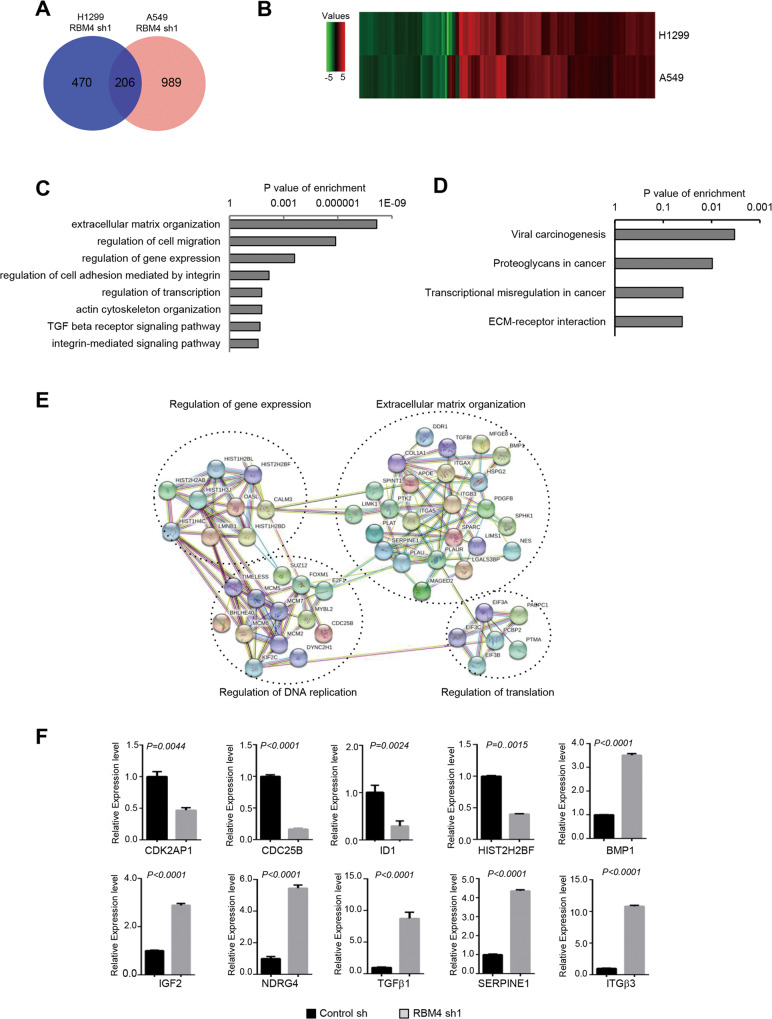
Global identification of genes that are regulated by depletion of RBM4 in senescence. **A** Venn diagram showing differentially expressed genes overlapping between H1299 and A549 cells with stable knockdown of RBM4 by RNA seq. **B** Heatmap depicting levels of significantly changed genes in H1299 and A549 cells with stable knockdown of RBM4. **C** Gene ontology of the overlapped genes in biological processes. **D** KEGG analysis of overlapped differentially expressed genes. **E** Functional association network of RBM4-regulated genes in (**A**) were analyzed using the STRING database, and subgroups are marked according to their functions. **F** Validation of gene expression changes in RBM4-depleted H1299 cells by real-time RT-qPCR. The mean ± SD of relative fold changes from triplicate experiments were plotted with *P* values calculated by paired *t* test.

To systematically identify genes that are regulated by RBM4-depletion in senescence, we performed RNA-seq with both H1299 and A549 cells stably knocking down RBM4 or control respectively. We identified 1195 genes in A549 cells and 676 genes in H1299 cells that are significantly changed (>2-fold with q < 0.05). Subsequently, we compared the significantly changed genes in the two cell lines, and found 206 commonly altered genes that are potential targets for RBM4 during senescence (Fig. 4A, B). Unbiased gene ontology (GO) analysis of the overlapped genes revealed that these genes are significantly associated with ECM organization, regulation of cell migration, regulation of gene expression, regulation of cell adhesion mediated by integrin, regulation of transcription, actin cytoskeleton organization, TGFβ receptor signaling pathway, and integrin-mediated signaling pathway (Fig. 4C). Meanwhile, we performed KEGG analysis and found that interactions induced by depletion of RBM4 were focused on viral carcinogenesis, proteoglycans in cancer, transcriptional dysregulation in cancer, ECM-receptor interaction (Fig. 4D). In addition to analyzing the GO and KEGG enrichment of the overlapped genes changed in both of two cell lines, we also investigated the enrichment in the two cell lines separately. Similarly, ECM organization, regulation of gene expression, cell cycle regulation, TGFβ receptor signaling pathway, and so on are still the top enrichments by GO analysis (Fig. S3A, B), whereas ECM-receptor interaction and transcriptional dysregulation in cancer are critical pathways regulated by knockdown of RBM4 as well (Fig. S3C, D).

We next conducted network analysis using the STRING database to evaluate the interaction of these overlapped genes. Consistently, a number of RBM4-depletion regulated genes were also functionally connected into a densely linked network that includes genes in ECM organization, regulation of gene expression, regulation of DNA replication, and regulation of translation (Fig. 4E). To further validate the RNA-seq data experimentally and verify the role of depleted RBM4 in senescence, we chose some senescence related genes and performed the real time RT-PCR assay. As expected, all of the selected targets were validated (Fig. 4F and S3E). Collectively, many of the overlapped genes are enriched in ECM organization, and regulation of gene expression as accessed by GO, KEGG, and STRING analysis, suggesting that RBM4 might contribute to senescence through regulating the genes involved in these pathways.

### RBM4 regulates senescence through modulating the expression level of SERPINE1

SERPINE1 encoding Plasminogen activator inhibitor-1 (PAI-1) was one of the top hits in the RNA-seq data that was upregulated upon RBM4 depletion in both of two cell lines (Fig. 4F and S3E). In addition to regulating fibrinolysis, SERPINE1 also directly contributes to cellular senescence [24]. Therefore, we next sought to determine whether RBM4 participates in senescence through regulating SERPINE1.

**Fig. 5:**
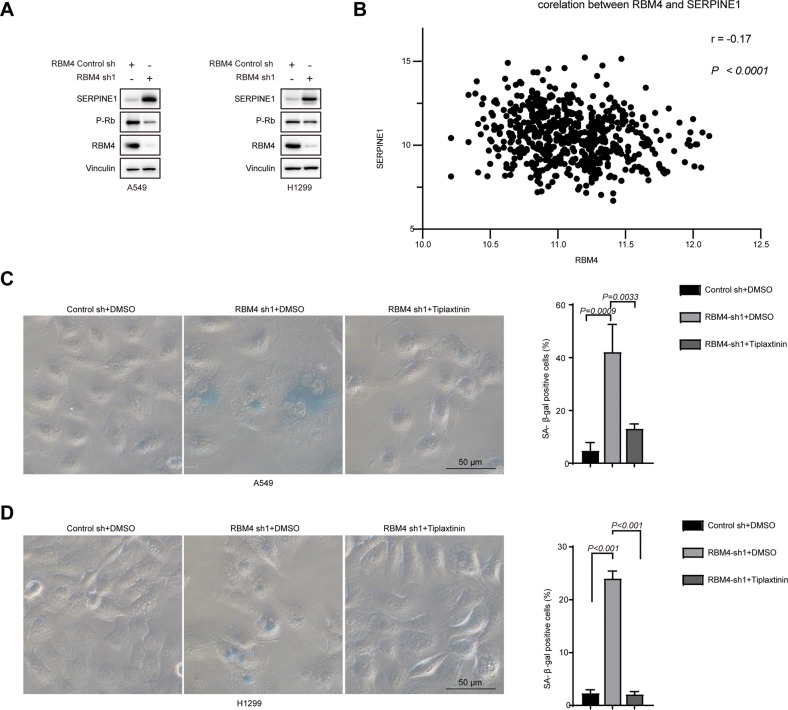
RBM4 regulates senescence through modulating the expression level of SERPINE1. **A** The protein levels of SERPINE1 in A549 and H1299 cells with RBM4 depletion were examined using a western blot assay. **B** Correlation analysis between RBM4 and SERPINE1 expression was performed using TCGA data, and the Pearson correlation coefficient was calculated. **C** β-gal staining of RBM4-depleted A549 cells with or without treatment of tiplaxtinin. Three experiments were carried out with mean ± SD of β-gal positive cells plotted (*P* values were determined by One-way ANOVA with Dunnett multiple comparisons). **D** β-gal staining of RBM4-depleted H1299 cells with or without treatment of tiplaxtinin. Three experiments were carried out with mean ± SD of β-gal positive cells plotted (*P* values were determined by One-way ANOVA with Dunnett multiple comparisons).

We measured the protein level of SERPINE1 in RBM4 depleted cells. We observed that the level of SERPINE1 was endogenously activated upon knockdown of RBM4 as compared to the control in two different lung cancer cell lines (Fig. 5A). Consistently, we found that the expression levels of SERPINE1 was negatively correlated to the expression levels of RBM4 (Fig. 5B).

We next investigated whether depletion of RBM4 contributed to senescence through regulating SERPINE1. As expected, RBM4 depletion-induced senescence could be partially reversed by inhibition of SERPINE1 with tiplaxtinin (a SERPINE1 inhibitor) in lung cancer cells, as judged by SA-β-gal activity (Fig. 5C, D). Therefore, our data suggested that depletion of RBM4 might induce senescence through elevating SERPINE1.

### RBM4 regulates the expression level of SERPINE1 via miR-1244

**Fig. 6:**
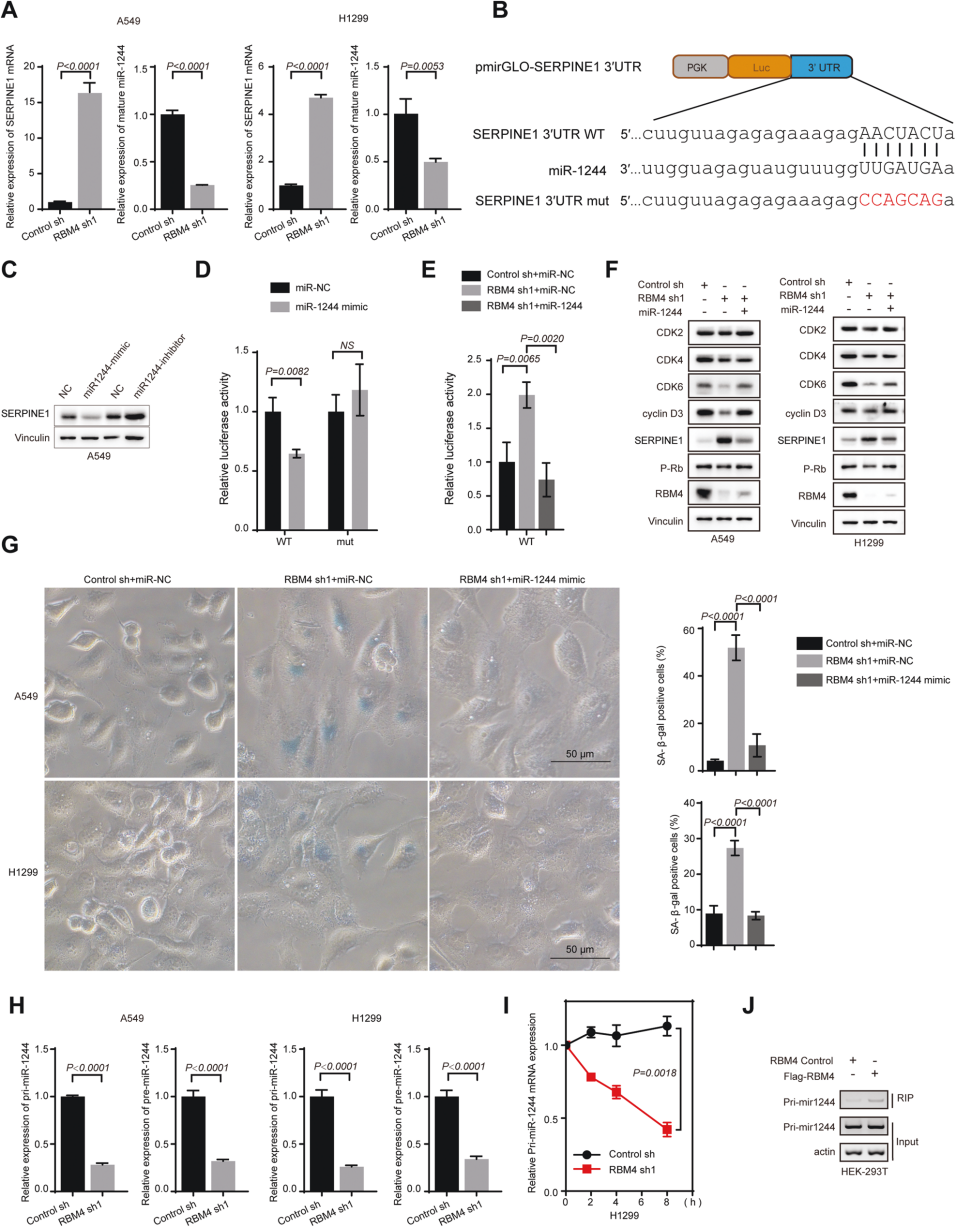
RBM4 regulates the expression level of SERPINE1 via miR-1244. **A** The expression of SERPINE1 and miR-1244 in RBM4-delepeted A549 and H1299 cells were determined using RT-qPCR. **B** Predicted binding sites for miR-1244 on SERPINE1 and a diagram depicting the construction of pmirGLO-SERPINE1-3′UTR-WT and pmirGLO-SERPINE1-3ʹUTR-mut plasmids. **C** A549 cells were transfected with miR-1244 mimics or miR-1244 inhibitor. The protein level of SERPINE1 was detected by a western blot assay. **D** The luciferase reporter pmirGLO-SERPINE1-3ʹUTR-WT/mut were co-transfected with miR-NC or miR-1244 mimic. Luciferase activity was detected 24 h after transfection using a dual-luciferase assay. The relative luciferase activities were determined by calculating the ratio of firefly luciferase activities over renilla luciferase activities. Error bars are mean ± SD and *P* values were determined by One-way ANOVA with Dunnett multiple comparisons. **E** The luciferase reporter pmirGLO-SERPINE1-3ʹUTR-WT was transiently transfected into RBM4 stably depleted H1299 cells with miR-NC or miR-1244 mimic. **F** Protein levels of CDK2, CDK4, CDK6, cyclin D3, SERPINE1, P-Rb and RBM4 were measured in RBM4 depleted A549 and H1299 cells with miR-1244/NC mimics. **G** β-gal staining of RBM4-depleted A549 and H1299 cells with miR-1244/NC mimics. Error bars indicate the mean ± SD. **H** The expression of pri-miR-1244 and pre-miR-1244 in RBM4-delepeted A549 and H1299 cells were determined using RT-qPCR. **I** RBM4-depleted H1299 cells were treated with actinomycin D as indicated times and the mRNA expression levels of pri-miR-1244 were examined using RT-qPCR. *P* values were determined using two-way repeated measures ANOVA. **J** Binding of pri-miR-1244 with RBM4 is determined by RNA-immunoprecipitation in HEK-293T cells expressing FLAG-RBM4.

We next sought to investigate how RBM4 regulates the expression of SERPINE1. We analyzed the RNA-seq data and found that a precursor miRNA (microRNA-1244-2) was decreased upon RBM4-depletion along with increased SERPINE1. We confirmed that the level of SERPINE1 was increased and the mature miRNA level of miR-1244 was suppressed upon depletion of RBM4 as compared to the control in two different lung cancer cell lines (Fig. 6A and Fig. S4A). By using TargetScan analysis, we revealed that the 3ʹ-UTR of SERPINE1 contained the potential binding sites of miR-1244 (Fig. 6B). To validate this result, we applied the miR-1244 mimic and miR-1244 inhibitor in A549 and H1299 cells. As expected, the miR-1244 mimic decreased the protein level of SERPINE1, whereas the miR-1244 inhibitor increased the protein level of SERPINE1 (Fig. 6C and Fig. S4B). Additionally, the results obtained from the luciferase reporter assay indicated that miR-1244 directly binds to the 3′-UTR of SERPINE1 to inhibit the expression of SERPINE1, while mutation of the miR-1244 binding site in SERPINE1 abolished such suppression (Fig. 6D). Moreover, miR-1244 mimic could suppress the RBM4 depletion-induced upregulation of SERPINE1 induced as judged by luciferase reporter assay (Fig. 6E).

We next determined whether the miR-1244/SERPINE1 was responsible for RBM4 depletion induced senescence. We found that the application of the miR-1244 mimic at least partially rescued the senescence in RBM4 depleted cells, as judged by the corresponding changes at the protein levels of senescence-related markers (e.g. p-RB, CDK2, CDK4, CDK6 and Cyclin D3) and SA-β-gal activity in both A549 and H1299 cells (Fig. 6F, G). Taken together, our data suggest that depletion of RBM4 increased the level of SERPINE1 through downregulating miR-1244, thereby inducing senescence and inhibiting growth.

To further investigate how RBM4 regulates the expression of miR-1244, we analyzed the levels of pri-miR-1244 and pre-miR-1244 in RBM4 depleted cells by RT-qPCR, and found that the level of pri-miR-1244 was significantly decreased (Fig. 6H). We also examined whether RBM4 modulates the mature miRNA level of miR-1244 through regulating the stability of pri-miR1244. We treated RBM4-depleted or control cells with actinomycin D, an RNA synthesis inhibitor, and found that knockdown of RBM4 significantly promoted the degradation of pri-miR1244 (Fig. 6I). In addition, we performed RNA-immunoprecipitation (RIP) assay, and revealed that RBM4 could indeed interact with pri-miR1244 (Fig. 6J and Fig. S4C). Taken together, our data suggest that depletion of RBM4 downregulates miR1244 by promoting the pri-miR1244 degradation, thus increasing the level of SERPINE1, which induces senescence and inhibits cancer cell growth.

## Discussion

**Fig. 7:**
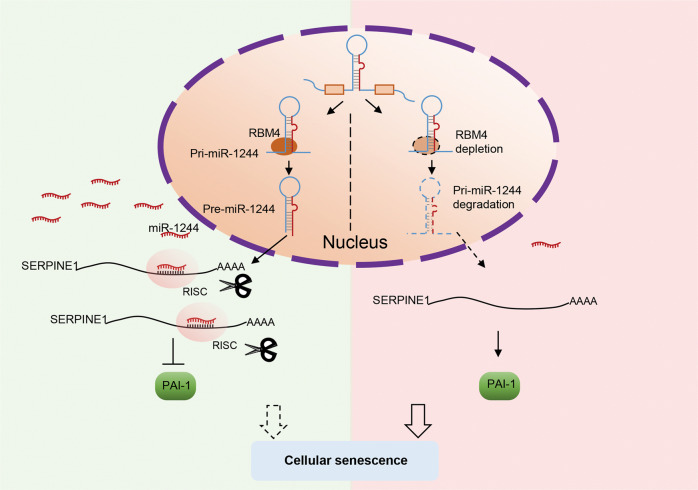
Schematic diagram of the mechanism that RBM4 regulates cellular senescence in cancer. RBM4 binds to pri-miR1244, maintaining the level of miR1244 and inhibiting SERPINE1 expression, whereas depletion of RBM4 reduces the level of miR-1244 by promoting degradation of pri-miR1244, thus increasing SERPINE1 expression and inducing subsequent senescence.

Accumulating evidence has implied that senescent cells could accumulate in precancerous lesions to provide a barrier for cancer cell proliferation, thus to inhibit cancer progression [25, 26]. Here we showed that RBM4 was inversely correlated to senescence. Our results demonstrated that depletion of RBM4 promoted multiple cancer cell senescence, as judged by increased SA-β-gal activity, resulting in cell cycle arrest, inhibition of cell proliferation, and alterations at protein levels of senescence related markers. Mechanistically, our data, for the first time, showed that depletion of RBM4 played critical roles in elevating the level of SERPINE1 to promote cancer cell senescence, thereby inhibiting cancer progression. We identified that miR-1244 bound to the 3′-UTR of SERPINE1 to suppress the expression of SERPINE1, whereas depletion of RBM4 decreased the expression of miR-1244 by promoting pri-miR1244 degradation (Fig. 7). Collectively, these data provide a novel mechanism of RBM4, which can affect cancer progression through post-transcriptional regulation.

MicroRNAs (miRNAs) are small non-coding RNAs with 20–24 nucleotides, which regulate the expression of thousands of genes at posttranscriptional level. The biogenesis of miRNA involves transcription of miRNA genes, processing of primary miRNA transcripts by Dicer proteins into mature miRNAs, and loading of mature miRNAs into Argonaute proteins to form miRNA-induced silencing complex (miRISC). By targeting complementary mRNA, miRISC silence gene expression through mRNA destabilization and translational repression, which involved in the regulation of cellular development, proliferation, differentiation, apoptosis, metabolism and cellular senescence [27]. It has been previously reported that miR-1244 plays an essential role in the regulation of cell proliferation and cisplatin resistance [28–30]. RBM4 could directly interact with Ago2 and recruit Ago2 to suppress translation of target mRNAs during muscle cell differentiation [14, 20]. Here, we found that RBM4 could directly bind to pri-miR-1244 and maintain the stability of pri-miR-1244. Depletion of RBM4 reduced the level of miR-1244 by promoting the degradation of pri-miR1244, thus increasing the expression of SERPINE1. We demonstrated that the 3’UTR of SERPINE1 harbored the binding sites of miR-1244, which was further confirmed by dual-luciferase reporter assay. Taken together, depletion of RBM4 reduced the level of miR-1244, increasing the mRNA expression of SERPINE1 and inducing subsequent senescence.

Our previous study have reported that RBM4 suppresses tumorigenesis through modulating the alternative splicing of cancer-related genes [16, 17]. In the current study, we also conducted RNA-seq and tried to identify the senescence-related alternative splicing that were regulated by depletion of RBM4. However, the RBM4-regulated splicing events were not able to enrich in senescence, suggesting that RBM4 might not participate in senescence regulation through directly modulating alternative splicing. Nevertheless, we cannot exclude the possibility that RBM4 might control the splicing of certain genes, thereby indirectly regulating senescence.

We have previously shown that RBM4 might function as a potential tumor suppressor to inhibit cancer progression [16]. However, in this study, our data demonstrated that loss of RBM4 promoted cell senescence to inhibit tumorigenesis, which appears to be controversial to our previous work. The possible reason for this might be that the level of RBM4 needs to be finely regulated. Both more and less of RBM4 could suppress cell proliferation through different mechanisms. Similarly, the tumor suppressor PTEN could control DNA repair, and cell cycle to inhibit cancer progression [31, 32]. Meanwhile, loss of PTEN could promote cellular senescence to suppress tumorigenesis [33]. In addition, PTEN loss induced cellular senescence has been recognized as a therapeutic target [34]. Therefore, targeting the senescence that is induced by depletion of RBM4 might also represent therapeutic potential for cancer treatment.

In conclusion, we revealed that RBM4 controlled cellular senescence to regulate cancer progression via miR1244/SERPINE1 axis. RBM4 ablation induced senescence and inhibited cancer cell progression both in vitro and in vivo. This finding not only offers a new avenue for targeting RBM4 in cancer therapy, but also provides novel mechanistic insights of the RNA binding protein-regulated senescence.

## Supplementary information

Supplemental figures

Original Data File

checklist

Supplemental Table

## Data Availability

RNA-seq data in this study have been deposited in Gene Expression Omnibus of NCBI with the accession code GSE194307. The authors declare that all the data supporting the findings of this study are available within the article and its Supplemental information files.
