# Supplementary material for: RBM4 regulates cellular senescence via miR1244/SERPINE1 axis
Source: Cell Death Dis. 2023 Jan 13;14(1):27. doi: 10.1038/s41419-023-05563-z (PMC9839707; doi:10.1038/s41419-023-05563-z)
Supplement: Supplementary file 1 — Supplemental figures [file 41419_2023_5563_MOESM1_ESM.pdf]

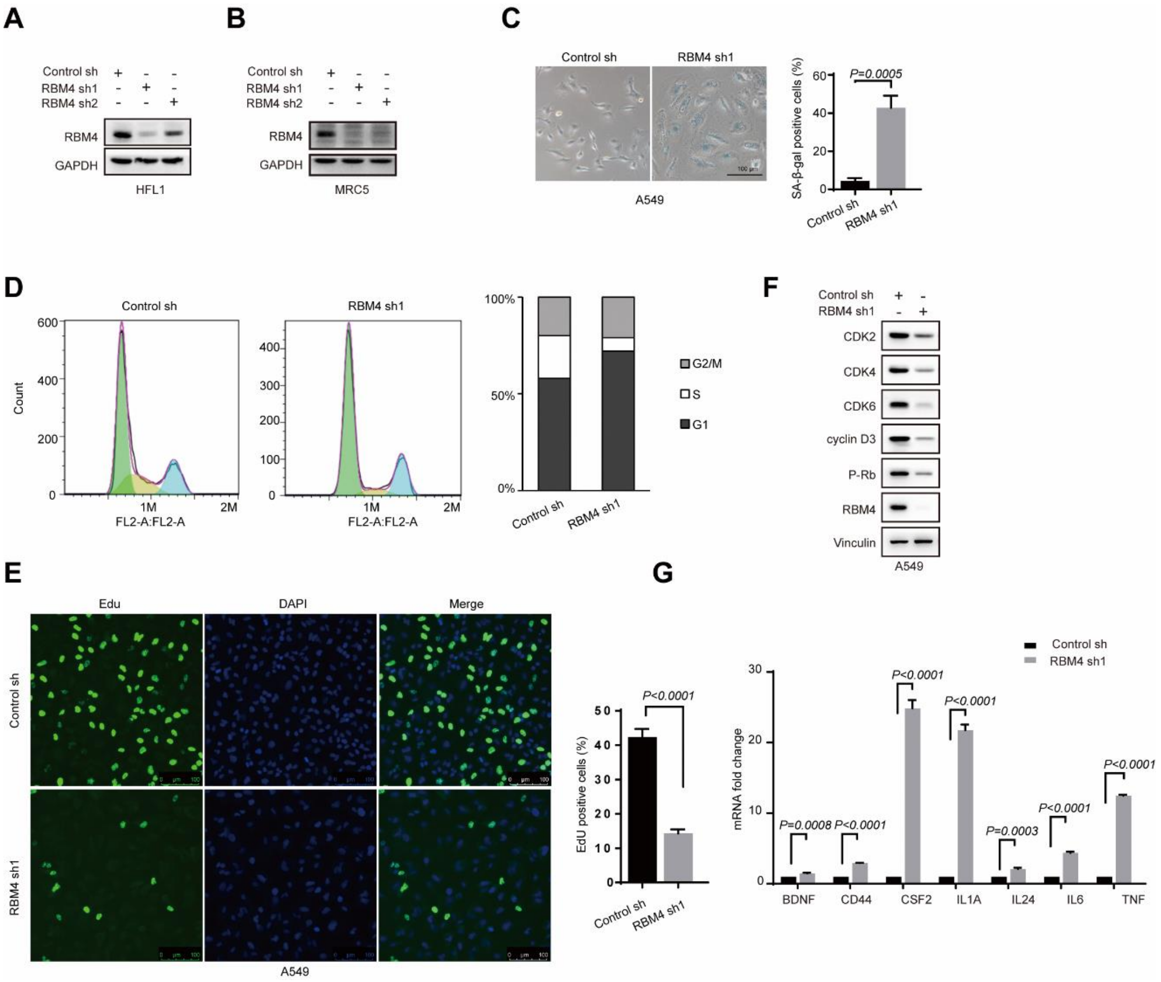

Supplementary Fig. 1 Reduction of RBM4 induces senescence in MRC5 and A549 cells. (A) Protein levels of RBM4 was measured in HFL1 cells with stable knockdown of RBM4. (B) Protein levels of RBM4 was measured in MRC5 cells with stable knockdown of RBM4. **(C)** β-gal staining of A549 cells with stable knockdown of RBM4. **(D)** Cell cycle analysis calculated the distribution of the cells in G1, S and G2/M phases in RBM4 depleted A549 cells. **(E)** The proliferative abilities of stably RBM4-depleted A549 cells were measured with an EdU staining assay. **(F)** The protein levels of RBM4, CDK2, CDK4, CDK6, cyclin D3 and p-Rb in A549 cells with RBM4 depletion were examined using a western blot assay. **(G)** The mRNA expression levels of senescence associated pro-

inflammatory genes including interleukin 1A, 6, 24 TNF, CD44 and BNDF in RBM4-depleted A549 cells were examined by qRT-PCR.

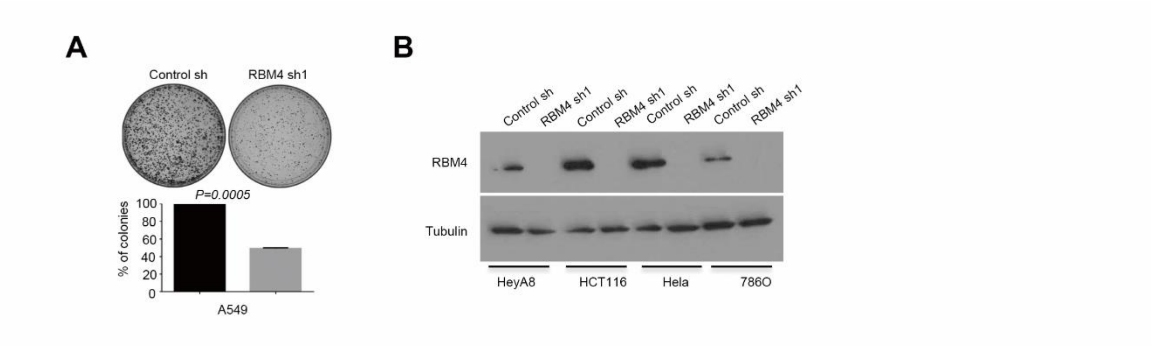

Supplementary Fig. 2 Reduction of RBM4 inhibits tumorigenesis in A549 cells. (A) Colony formation assays of RBM4-depleted A549 cells were performed. **(B)** Protein levels of RBM4 was measured in HeyA8, HCT116, Hela and 786O cells with stable knockdown of RBM4.

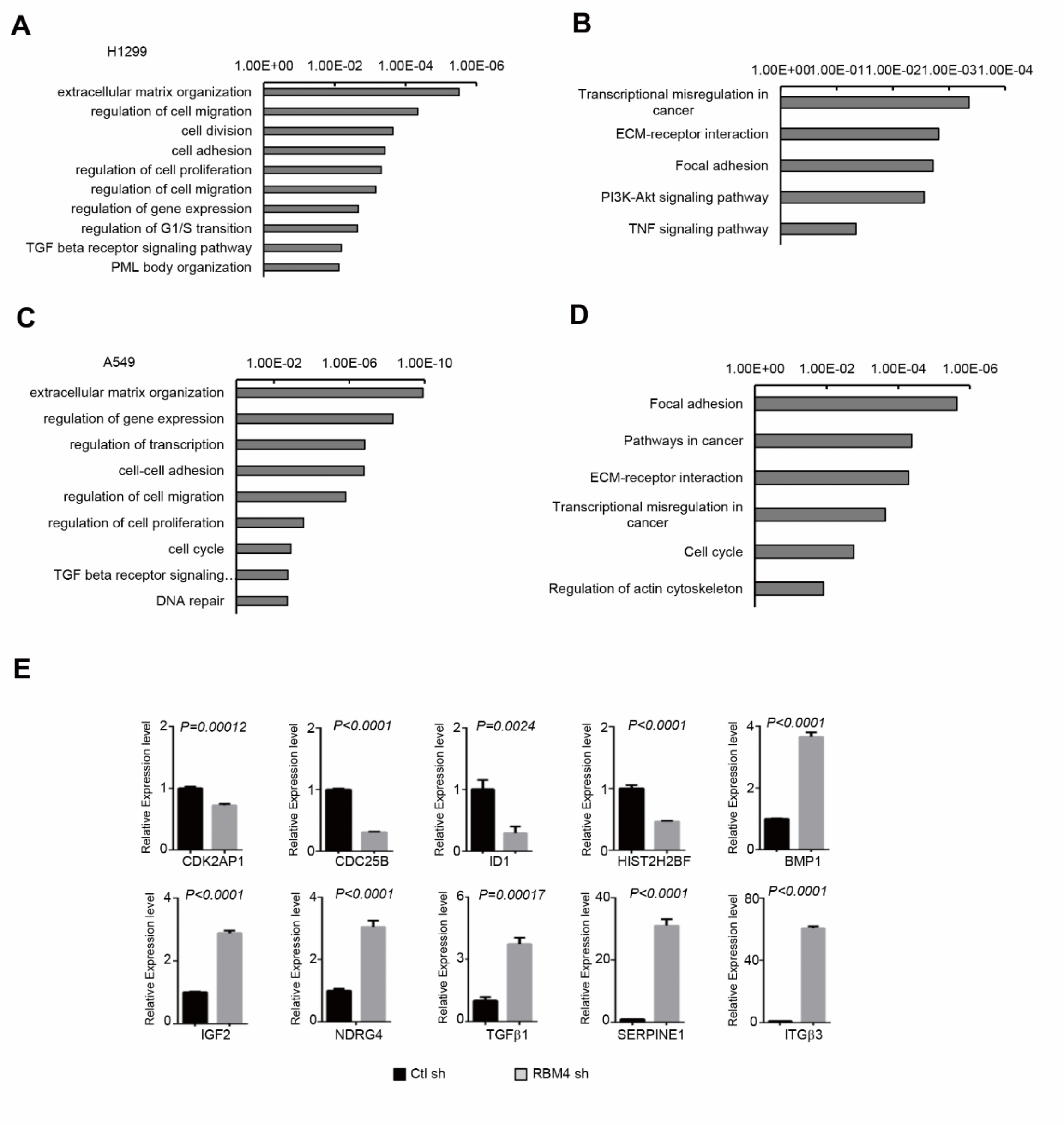

Supplementary Fig. 3 Global identification of genes that are regulated by depletion of RBM4 in senescence. (A) Gene ontology of differentially expressed genes in H1299 cells with stable knockdown of RBM4 by RNA seq. **(B)** KEGG analysis of differentially expressed genes in H1299 cells with stable knockdown of RBM4. **(C)** Gene ontology of differentially expressed genes in A549 cells with stable knockdown of RBM4 by RNA seq. **(D)** KEGG analysis of differentially expressed genes in A549 cells with stable knockdown

of RBM4. **(E)** Validation of gene expression changes in A549 cells by qRT-PCR. The mean ± SD of relative fold changes from triplicate experiments were plotted with p values calculated by paired t-test.

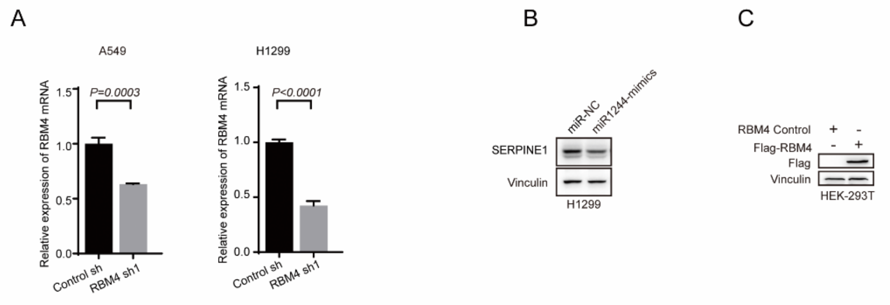

Supplementary Fig. 4 RBM4 regulates the expression level of SERPINE1 via miR-1244. (A) The expression of RBM4 in RBM4-delepeted A549 and H1299 cells were determined using RT-qPCR. **(B)** H1299 cells were transfected with miR-1244 mimics. The protein level of SERPINE1 was detected by a western blot assay. **(C)** The expression of FLAG-RBM4 in HEK-293T cells were determined by a western blot assay.
