# Supplementary material for: RBM4 regulates cellular senescence via miR1244/SERPINE1 axis
Source: Cell Death Dis. 2023 Jan 13;14(1):27. doi: 10.1038/s41419-023-05563-z (PMC9839707; doi:10.1038/s41419-023-05563-z)

Figure1H(H1299)

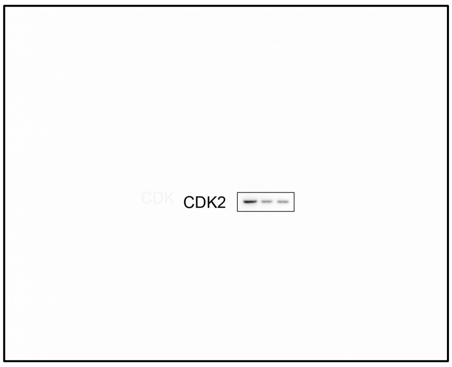

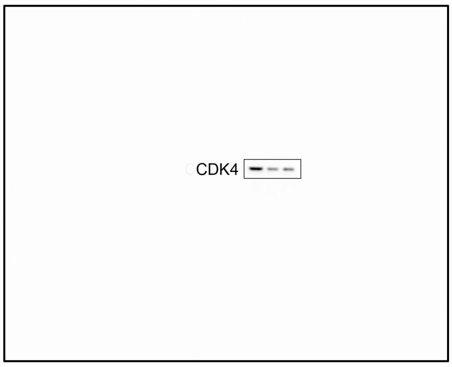

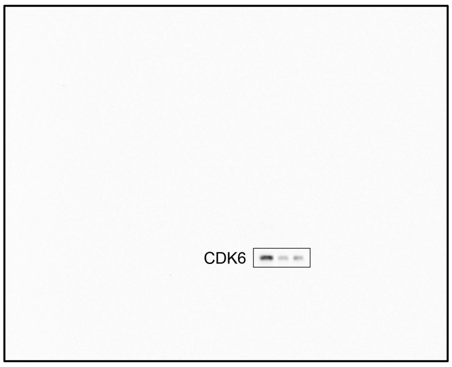

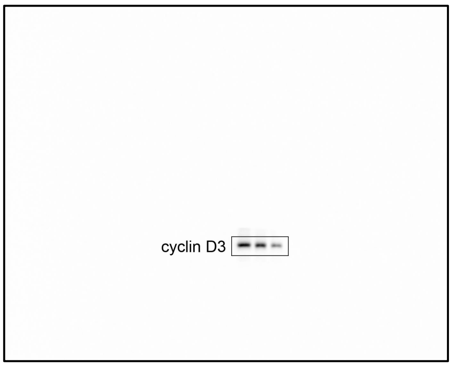

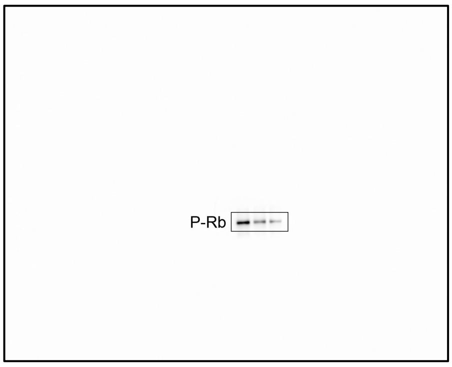

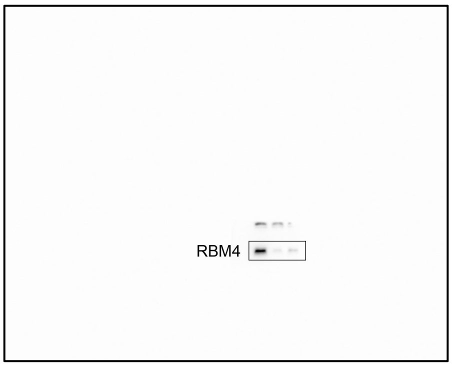

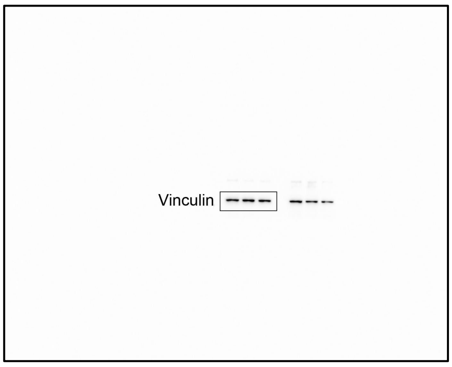

Figure5A
(A549)

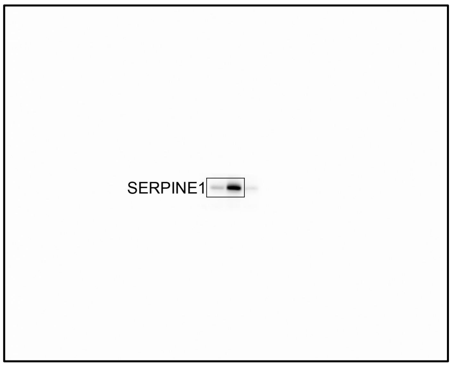

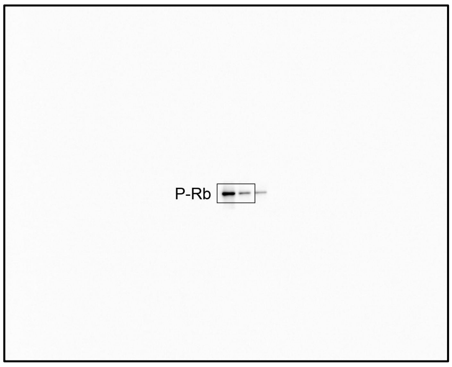

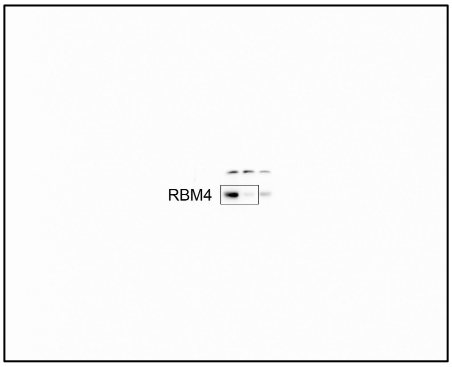

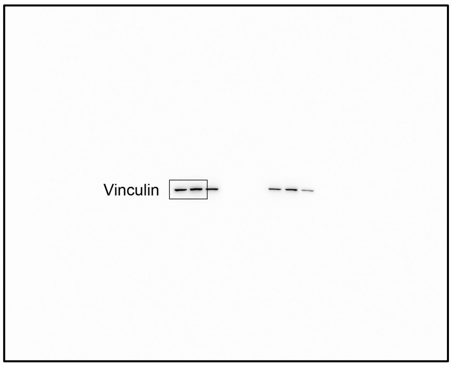

(H1299)

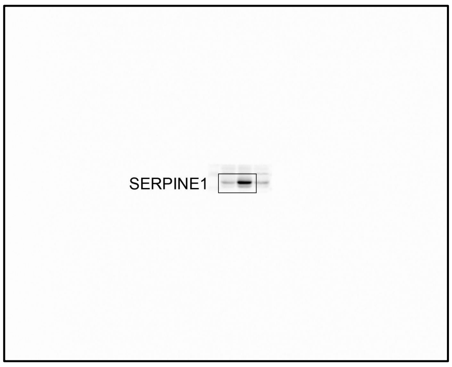

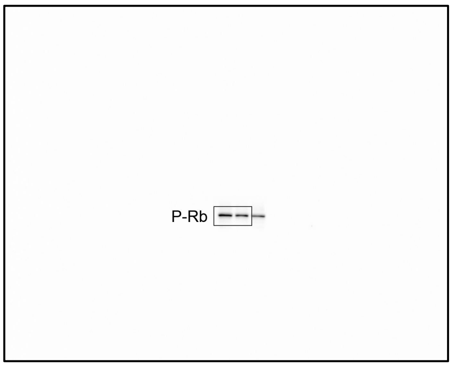

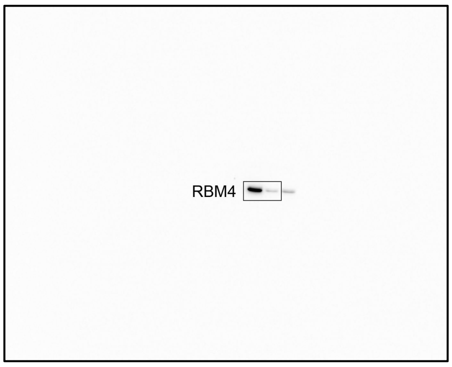

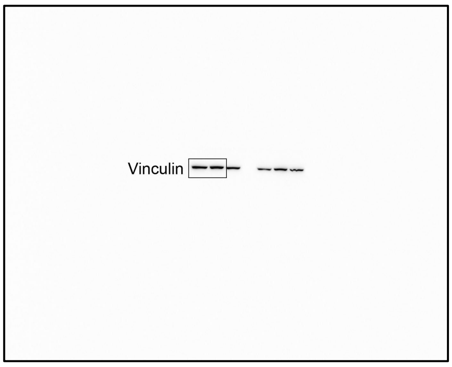

Figure6C(A549)

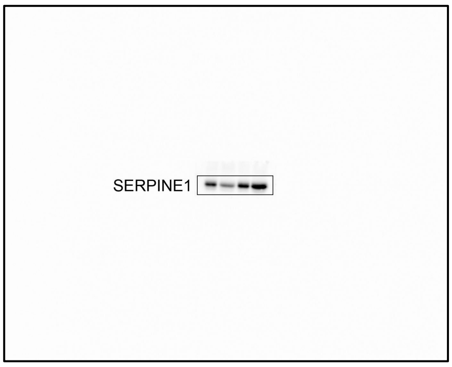

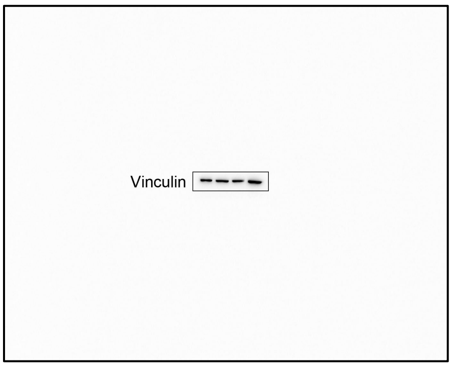

Figure6F

(A549)

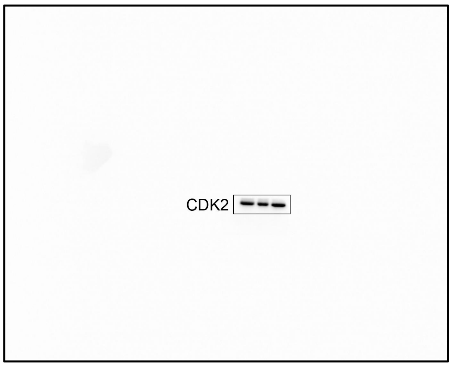

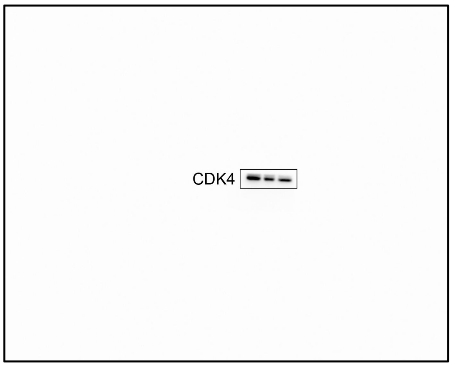

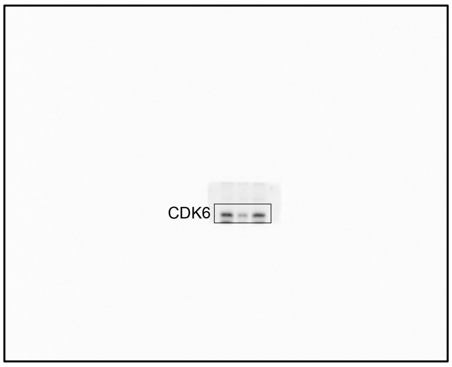

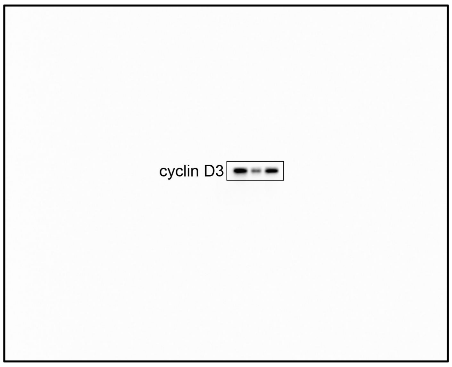

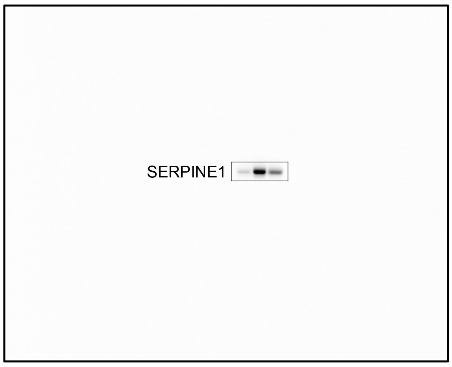

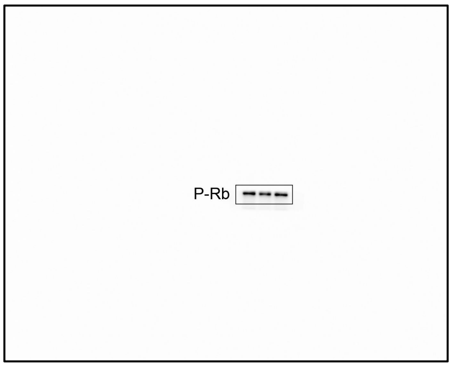

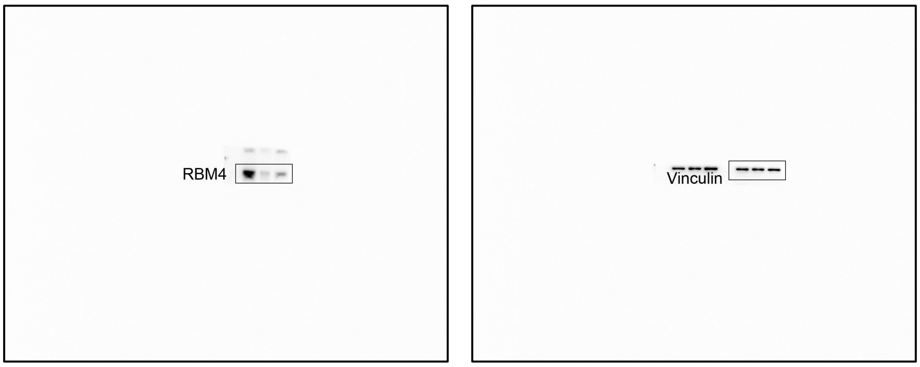

(H1299)

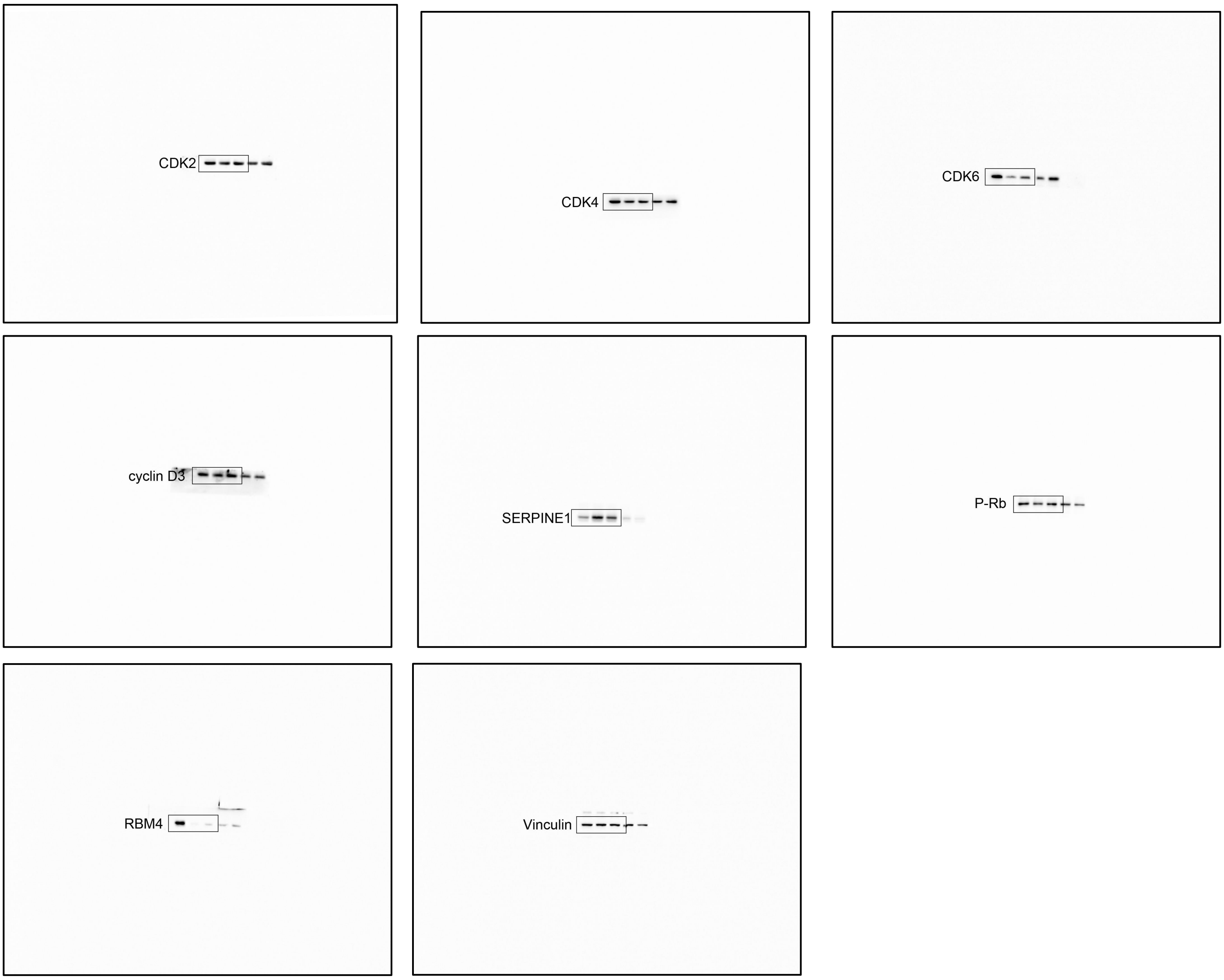

FigureS1A(HLF1)

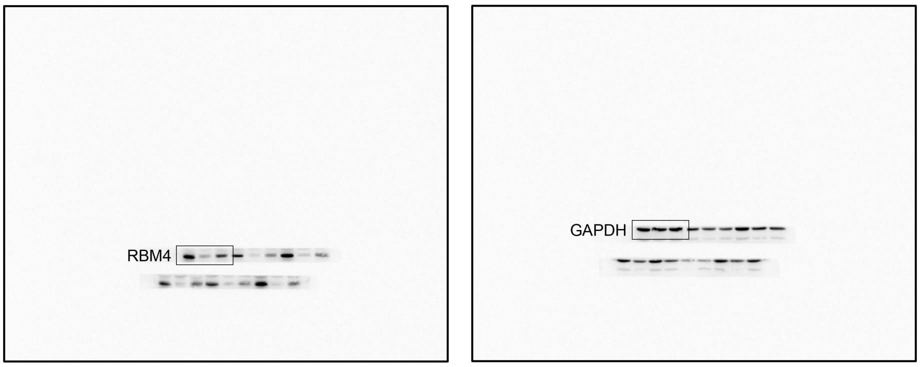

FigureS1B (MRC5)

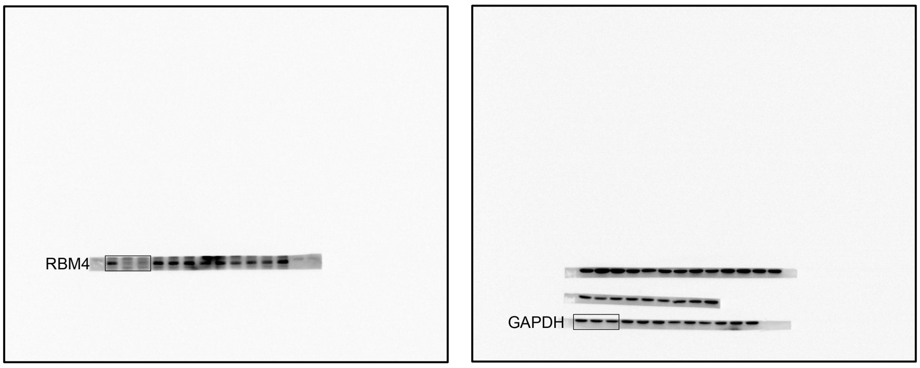

FigureS1F(A549)

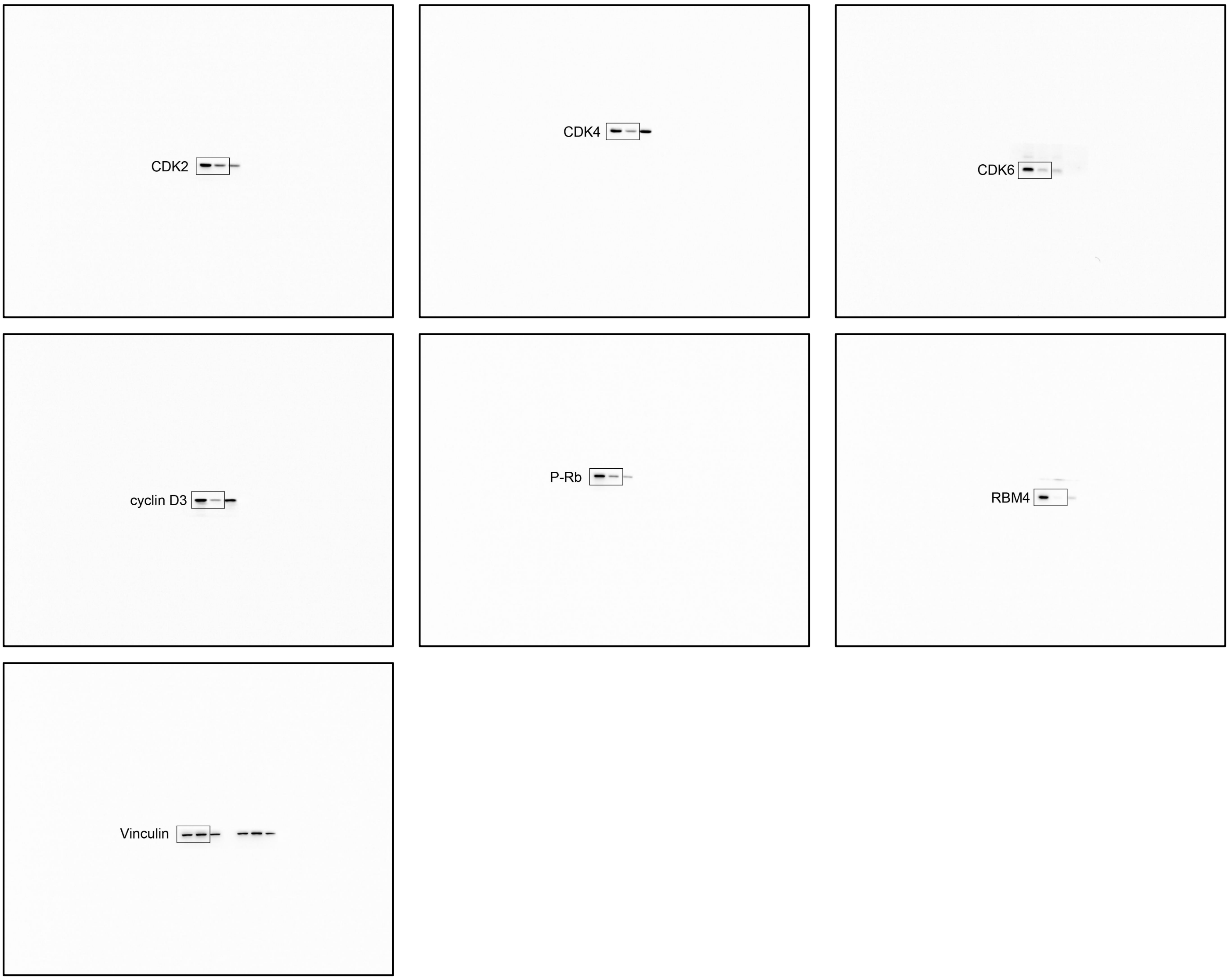

FigureS2B(HeyA8、HCT116、Hela、786O)

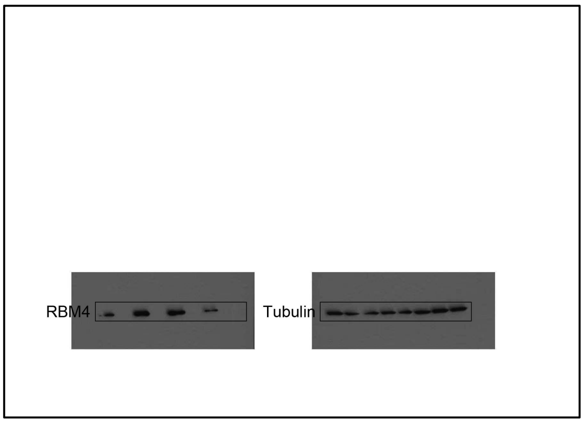

FigureS4B(H1299)

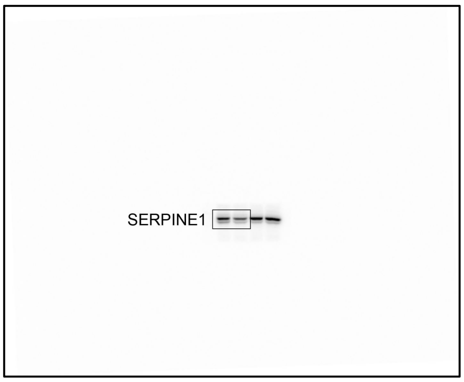

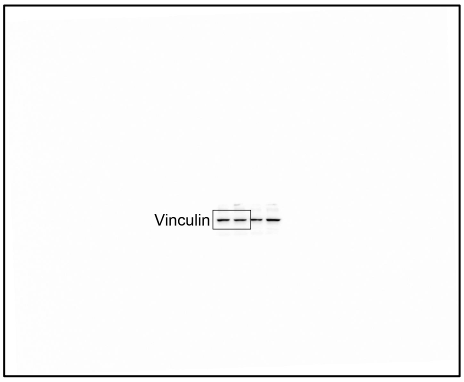

FigureS4C(HEK-293T)

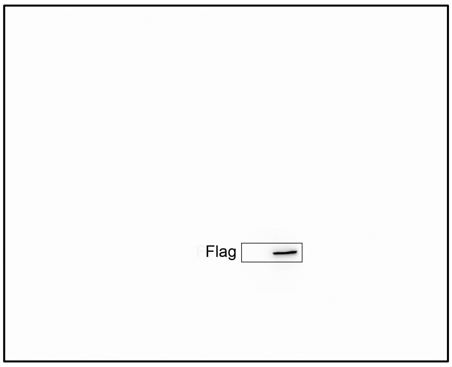

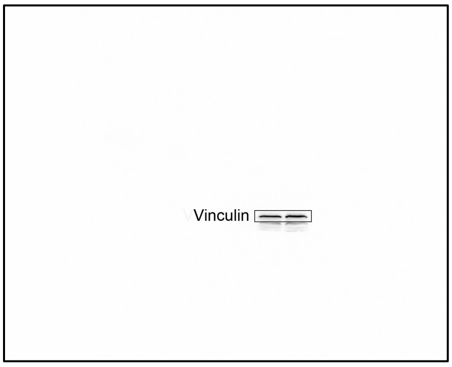

Supplement: Supplementary file 2 — Original Data File [file 41419_2023_5563_MOESM2_ESM.pdf]
